# Decoding inequalities in the journalistic field: A theoretical approach to how symbolic violence shapes journalistic career trajectories

**DOI:** 10.1177/14648849261416555

**Published:** 2026-01-16

**Authors:** Kim Löhmann, Phoebe Maares

**Affiliations:** 27258 University of Vienna, Austria; University of Tübingen, Germany

**Keywords:** diversity, inequalities, field theory, symbolic violence, intersectionality, journalism

## Abstract

Achieving diversity is a fraught process in the journalistic field, with persistent inequalities shaping the experiences of marginalized journalistic actors. By combining Bourdieu’s field theory and Crenshaw’s concept of intersectionality, we offer a conceptual framework tracing how marginalized journalistic actors experience symbolic violence and symbolic dominance in different journalistic fields around the globe. These experiences can stem from journalists’ lack of appropriate forms of capital, the “right” journalistic habitus and an understanding of the doxa shaping what journalism is: what it ought to look like and who can be considered a legitimate and authoritative member of a given journalistic field. We illustrate this conceptual framework using possible career trajectories of (marginalized) journalists to highlight various forms of symbolic violence experienced by (dis)empowered actors along the three main stages of journalistic careers—namely becoming a journalist, being a journalist and potentially leaving journalism later on.

## Introduction

Diversity in journalism, a widely explored topic, has been situated within larger debates surrounding democratic ideals of pluralism and social equality in the media. A rich body of research highlights the lack of diversity and persisting inequalities within journalism, observable in its actors, content, broader ownership and audience structures (e.g., Borchardt et al., 2019). Scholars chiefly critique the marginalization^1^ of disadvantaged actors in journalism, problematizing the exclusion of diverse perspectives from public discourse while simultaneously reproducing the dominant, and oftentimes exclusionary, norms and values that shape the journalistic profession and newsroom culture (Banjac, 2024b). Furthermore, journalistic socialization is centered around hegemonic ideals of what journalism ought to do, ultimately marginalizing perspectives that diverge from this normative path (e.g., Douglas, 2022; North, 2016). Hence, journalism has been described as dominated by the elite’s viewpoints, turning the field into a “treacherous space” (Banjac, 2024b: 359) for marginalized journalists. In the face of growing societal pressures, newsrooms especially, but not exclusively, in the Global North have begun to address these debates by increasing efforts to implement diversity policies (Assmann and Eckert, 2024; Bodinger-de Uriarte and Valgeirsson, 2015). Consequently, news outlets explicitly seek “diverse” applicants in hiring processes, which can result in tokenism and reductionist approaches to complex identities (Kanter, 1997; Lachover, 2022).

While these measures can increase diversity within newsrooms, they tend to force marginalized journalists into being spokespeople for entire communities, serving as ornamental public representatives used to showcase newsroom diversity (Awad-Cherit, 2008; Carter and Ferrucci, 2024; Thomas and Nolan, 2024). Studies on long-term employment of marginalized journalists, however, have shown that persistent discrimination and cultural norms push marginalized journalists to leave the profession at higher rates than their non- or less marginalized colleagues (Carter et al., 2019). This shows how social inequalities shape the experience of marginalized journalists in the dominant professional culture, emphasizing the necessity to engage more meaningfully with the concept of diversity in journalism and journalism research (Douglas, 2022).

This article reframes debates on journalistic diversity by examining it as a manifestation of deeply rooted, historically grown social inequalities, discriminatory practices and intersecting power dynamics that are difficult to address with diversity policies alone (Thomas and Nolan, 2024). Combining Bourdieu’s (1977, 1998) field theory with the concept of intersectionality (Collins and Bilge, 2020; Crenshaw, 1991), we argue that structural inequalities can shape professional experiences and career trajectories of marginalized journalists within traditional newsrooms around the world along three main stages—namely becoming a journalist, being a journalist and breaking away from journalism. Put differently, this article decodes and conceptually traces what happens to “those who come to represent ‘diversity’” (Mirza, 2006: 101) in journalism and highlights the conditions that shape their experiences. Rather than focusing on a specific national context or actor category, this conceptual framework combines field theory and intersectionality to illuminate power dynamics within journalism and their implications for individual careers and the field’s overall diversity. At the same time, it does not adopt a universalist approach to journalism but instead accounts for the diversity of journalistic cultures around the globe (Deuze and Witschge, 2018).

## Intersectional inequalities in representation

Inequalities in journalism have often been framed within broader debates on diversity and the importance of representation in fostering societal and political participation (Malik, 2014; McQuail, 1992). From a normative standpoint, journalism as an institution can be characterized as a mirror of society, “representing or reflecting the prevailing differences of culture, opinion, and social contributions of the population as a whole” (McQuail, 1992: 144). These differences across religion, ethnicity, race, geography, gender, sexuality, age, physical ability and social standing, and the intersections thereof, serve as markers of diversity (Crenshaw, 1989). However, journalism can be understood as a discursively constructed institution, meaning that members of the journalistic field discursively negotiate the prevailing societal norms, values and epistemologies shaping journalists’ professional environment and content (Hanitzsch and Vos, 2017). In doing so, they reproduce power structures within broader society such as racism, sexism and classism (Banjac, 2024b), contributing to systemic inequalities and discrimination and undermining true diversity.

Nevertheless, calls for greater diversity within media organizations are increasingly more pronounced. Disempowered journalists have begun to push for structural change through protests or open letters to editorial boards demanding institutional reforms (Douglas, 2022). In response, media organizations and journalism associations have focused on obtaining diversity within the journalistic workforce (Bodinger-de Uriarte and Valgeirsson, 2015). However, ethnic and race studies have shown that fostering diversity, for many organizations, is merely a performative response to society’s growing awareness of inequalities. Diversity has become more of a measure of institutional performance and economic potential than an impactful initiative generating change and democratic representation (Ahmed, 2007). In this sense, diversity is often framed as a managerial strategy, valued “as if it was a human resource” (Ahmed, 2007: 604) or “moral good in itself” (Mirza, 2006: 101), something economically desirable to be obtained and portrayed rather than a means of fostering genuine inclusivity, equality and moral righteousness. Scholars argue that this focus on diversity as a managerial or performance goal conceals the continuation of systematic inequalities and oppression, as it centers only on the image of being a “diverse” organization (Ahmed, 2007).

However, most diversity debates within news organizations tend to center around the experiences of specific collective identities that are in the minority or marginalized within their respective national contexts, for example, Black communities in British news media (Douglas, 2022), female journalists in India (Rao and Rodny-Gumede, 2020) or Indigenous journalists in Australia (Thomas and Nolan, 2024). The ramifications of such oversimplified approaches to diversity along singular identities are plentiful, as they dismiss the intersection and convergence of different identity markers, which result in different inequalities and expressions of oppression. As Collins and Bilge (2020) argue, the perceived membership of an individual to a certain group can make people vulnerable to certain forms of bias (e.g., racism or sexism). Thus, when journalists belong to several different groups confronted with different biases, their identity markers intersect, creating multi-dimensional and complex social identities and experiences that underlie inequalities and under- or misrepresentation in journalism. For instance, while the number of female journalists is increasing in many newsrooms, women of color, especially in leadership positions, remain underrepresented and barriers to lasting change persist (Macupe, 2025; Rao and Rodny-Gumede, 2020). Thus, establishing diversity is not only a matter of overcoming or succumbing to existing power inequalities but also pertains to how (marginalized) actors are socialized within the social context of a given field, their experiences and how these factors relate to other actors occupying the same space.

## A field theoretical perspective on power and structural inequalities

To understand the power dynamics that shape journalistic diversity—including its construction and continuity—and the relational stratification of inequalities, we adapt field theory (Bourdieu, 1977, 1998) and the concept of intersectionality, which was originally introduced to highlight the unique experiences of Black women but has since been extended to understand marginalized experiences more broadly (Collins and Bilge, 2020).

Field theory offers a framework to analyze society on both the structural and individual levels through the interplay of different concepts. From this theoretical perspective, journalism can be understood as a social field, which is, like society at large, stratified along different resources (*forms of capital*) and power dimensions, allowing some actors to occupy more powerful positions while marginalizing or disempowering others (Bourdieu, 1984; Collins and Bilge, 2020). The field is further structured by “taken for granted” beliefs (*doxa*) and internalized dispositions (*habitus*), which are internalized by individual members of a field and exist in objective structures (Bourdieu, 1977). Both habitus and forms of capital account for intersections of social positions and domination beyond class, allowing field theory to be expanded by the concept of intersectionality (Masquelier, 2019; Webb et al., 2017). Intersectionality captures how an individual’s various social categories (e.g., class, race, gender) overlap and might serve as a foundation of social inequalities in broader society and the journalistic field more specifically (Bourdieu, 1993, 1998; Byerly et al., 2023; Collins and Bilge, 2020). Thus, power relations in the field are not exclusively shaped by a privileged birth but also by the subsequent accumulation of money (economic capital), education and knowledge (cultural capital), social networks (social capital), recognition (symbolic capital) (Bourdieu, 1986) and the intersections thereof (Crenshaw, 1991). The unequal stratification of these resources in society serves as the basis of social inequalities, marginalization and intersectional power relations within the field, shaping an actor’s relative position, influence (Collins and Bilge, 2020) and capabilities to garner symbolic capital (Bourdieu, 1989).

The concept of habitus bridges the individual and societal levels (Bourdieu, 1977). At the individual level, habitus can be understood as someone’s “embodied social knowledge” (Sterne, 2003: 375), which they acquire through socialization, similar to the accumulation of embodied cultural capital (Moore, 2014). It encompasses individuals’ beliefs, values and manners developed through familial influences, professional experiences, or interactions with peer groups, as well as the capital accumulated over the course of their life (Maares and Hanusch, 2022). Habitus considers someone’s past while at the same time offering a horizon of possibilities (Bourdieu, 1977). However, these possibilities are not truly infinite but rather relational (Collins and Bilge, 2020): whereas a person with a well-educated, upper-class upbringing and an understanding of journalism as a vocation might have the “correct” habitus for many European journalistic fields, a person from a family with less engagement with journalism might find it harder to settle into the profession’s tacit rules (Vera-Zambrano and Powers, 2019).

On the societal level, habitus refers to the dominant predispositions in a field or institution, shaping the reproduction of power relations and the legitimacy of certain resources and relationships over others (Thomson, 2014). Thereby, journalists’ actions, behaviors and work are a reflection of the overall social context (Collins and Bilge, 2020) or the field’s genesis they are embedded within, as historical particularities of societies and their expressions of oppression shape individuals’ habitus (Bourdieu, 1993).

Habitus is also closely connected to a field’s doxa. As theories focusing on social relations, field theory and intersectionality assume that members of a field share certain beliefs of what is “true” and “taken for granted” (Bourdieu, 1977: 164). Consequently, doxa refers to classifying beliefs—on how to behave and which norms and values to adhere to—that have been naturalized over time in the field’s collective as well as in individuals, who thereby unconsciously contribute to social inequalities (Collins and Bilge, 2020). As a taken-for-granted belief, doxa is difficult to change unless it is specifically challenged—often by the dominant members of a field, especially in times of crisis—and enters the realm of discourse (Bourdieu, 1977: 169). Then, members of a field will stand in for orthodox positions (defending the belief) or heterodox positions (challenging it).

Lastly, coupled with the concept of doxa is the concept of *illusio*, which captures why members of a field invest themselves in the struggle of it (Bourdieu, 1998). Illusio relates to actors’ personal aspirations, motivations and goals and how they create and maintain meaning as members of a field. It is therefore mediated by, for example, class, ethnicity, geographic location and the experiences connected to these markers (Threadgold, 2018), which are at the same time informed by the already existing knowledge and predispositions about the field. Consequently, as participation in a field is shaped by actors’ embodied social history and knowledge, they might not even consider pursuing certain trajectories, viewing them as not “for the likes of us” (Bourdieu, 1990: 56). Actors can lose their illusio when structural circumstances are in extreme conflict with their practical sense, which can lead to disillusion or “stoic ataraxia,” defined by Bourdieu (1998: 77) as “the soul’s indifference, tranquility, or detachment.”

## Combating symbolic violence as a gateway to journalistic diversity

Journalistic careers are not solely the outcome of individual merit or effort but are also influenced by field effects and subsequent processes of legitimization and recognition by journalistic peers and professional gatekeepers (Collins and Bilge, 2020). Thus, a journalist’s ability to pursue a lasting and influential career is shaped by the accumulation of symbolic capital (Nölleke et al., 2023). Symbolic capital legitimizes which other forms of intersecting capital are worthy to accumulate by capturing which resources and properties are deemed valuable or powerful in a given field (Bourdieu, 1998; see also Collins and Bilge, 2020).

Journalistic capital—the symbolic capital unique to the journalistic field—is characterized by the knowledge and practices viewed as valuable or renowned within the field. It is often linked to peer recognition, from receiving prestigious journalistic awards to being perceived as more legitimate when, for example, working as an investigative journalist rather than a lifestyle reporter (Hovden, 2008; Nölleke et al., 2023; Schultz, 2007). Members of a field internalize these categories of distinction through their socialization and therefore know and recognize them, thus reproducing them at the same time (Bourdieu, 1984).

However, symbolic capital is unequally distributed in the field, creating a “hierarchy of worth and unworthiness” (Bourdieu, 2000: 241) or intersectional “privileges and punishments” (Byerly et al., 2023: 249) in which actors (re)produce field-specific capital to gain recognition or prestige, thus obtaining symbolic power. Bourdieu (1989: 22) described symbolic power as a relational power of “world-making”—the symbolic act of structuring the perception of the social world in the journalistic field, empowering and disempowering the actors within. The field’s structuring occurs across multiple domains of power, namely the *structural domain*, referencing fundamental societal structures (e.g., education or housing), the *cultural domain*, where some ideas or cultures are valued over others (e.g., doxa), the *disciplinary domain*, where rules and regulations are unevenly applied to different actor groups, and the *interpersonal domain*, the convergence of the three, shaping an individual’s positioning and symbolic power in society (Collins and Bilge, 2020).

Those with symbolic power (i.e., dominant actors) discursively construct the legitimate habitus for a journalist, the appropriate amounts of economic, cultural and social capital, as well as the inherent, and mostly unconsciously reproduced, dominant doxa of the journalistic field by shaping the perception of these field effects as valuable and symbolically powerful (Bourdieu, 1977).

In contrast, actors with less symbolic power in the field generally do not possess these renowned journalistic qualities or symbolic capital. For example, with journalists being primarily male globally (Ross Arguedas et al., 2024), gender has been conceptualized as negative symbolic capital by scholars, who argue that it can adversely affect the perceived value of women journalists’ skills and knowledge (Djerf-Pierre, 2007; Lucht and Batschelet, 2019). For women of color, their symbolic capital in the newsroom further decreases, as journalists deem “White, patriarchal establishments and norms to produce stories” (Banjac, 2024b: 363) more valuable than the heterodoxic approaches of women of color (Macupe, 2025), thereby naturalizing social inequalities (Collins and Bilge, 2020).

Social hierarchies emerging through the unequal stratification of symbolic capital and power are (re)produced and maintained through subtle and mostly invisible acts of symbolic violence. Unlike physical violence, symbolic violence is “gentle” (Bourdieu, 1977: 192), mostly “imperceptible and invisible to both those who wield it” (Banjac, 2024b: 364) and those who suffer from it. Journalists exerting symbolic violence do not necessarily do so consciously, but rather inflict harm through embodied dispositions such as their socialization (habitus) or internalized forms of (journalistic) symbolic capital. Likewise, journalists experiencing symbolic violence do not necessarily consciously accept it, but rather naturalize and misrecognize their suffering through internalized predispositions of what journalism ought to look like and what traits journalists should embody (Bourdieu, 1977; see also Banjac, 2024b). Symbolic violence manifests, for instance, when marginalized actors are pressured to minimize their diversity and conform to dominant field norms, such as the ideal of objectivity, or are confined to specific beats or topics due to their perceived identity markers (e.g., women to lifestyle sections, Black reporters to Black issues). Disempowered journalists often misrecognize other journalists’ perceptions of them being less accomplished or legitimate, attributing such presumptions to personal shortcomings rather than products of systematic power imbalances and structural inequalities (e.g., Collins and Bilge, 2020; Topić and Bruegmann, 2021). In doing so, they inadvertently help to naturalize and reproduce symbolic violence, becoming complicit in their own oppression (Bourdieu and Wacquant, 1992).

Individual experiences of symbolic violence shape journalists’ sense of belonging in the journalistic field, career trajectories, competitive opportunities (Collins and Bilge, 2020) and willingness to invest in the field long-term (illusio). Thus, when researching journalistic diversity, it is essential to add a temporal lens to the analysis, examining how journalists’ lived experiences and individual power throughout their entire careers shape the construction, discussion and potential restraint of diversity within the journalistic field.

Building on this premise, this conceptual article outlines and demonstrates the impact of field effects on (marginalized) journalists along three main journalistic career stages: becoming a journalist, being a journalist and (potentially) breaking away from journalism. Drawing on empirical research from across the world, this work highlights the necessity of approaching journalistic diversity more holistically, that is, from both a situational and a temporal perspective.

### Becoming a journalist

Aspiring journalists with a habitus that shares dispositions with more symbolically dominant journalists and who have accumulated the “right” amount and composition of different forms of capital can move through the process of becoming a journalist more easily. They experience the field and its taken-for-granted rules like a “fish in water” (Bourdieu and Wacquant, 1992: 127). In contrast, journalists with a diverging habitus might find it harder to embody the tacit rules and requirements of the journalistic profession and can thus be marginalized, facing inequalities in access and resources. For instance, journalists from disadvantaged backgrounds—such as people of color in post-apartheid South Africa or families in which underpaid, passion-driven work is uncommon—may struggle with the doxic rites of precarious early-career labor (Botma, 2012; Nölleke et al., 2022; Vera-Zambrano and Powers, 2019).

At this stage, the disproportionate distribution of symbolic power and the (often unconscious) enactment of symbolic violence become especially apparent, as disempowered actors struggle to meet entry requirements established by those in more powerful positions (*disciplinary domain of power*; see Collins and Bilge, 2020). For example, the growing expectation that journalists hold a graduate degree excludes individuals who cannot afford a university education or are unfamiliar with the process (Borchardt et al., 2019; Melki and Mallat, 2019). However, marginalized actors internalize what is deemed prestigious and legitimate within the field and strive for the same status markers, therefore unconsciously reinforcing the very structures that marginalize and oppress them (Schubert, 2014). Yet, their ability to meet those internalized requirements is often limited by the same structural constraints they seek to overcome (Collins and Bilge, 2020; Vera-Zambrano and Powers, 2019).

Individuals’ decisions to become journalists are often shaped by their habitus, which provides them with a practical sense of what is possible. This sense of possibility can be influenced, in part, by media consumption and representation, which shape an individual’s perceived fit within the profession (Lindell and Kas, 2024). The mediated portrayal of journalism can contribute to the “making of a social class” (Lindell and Kas, 2024: 22), including the journalistic class. In other words, depictions of journalists, whether in the news or popular culture, can shape an individual’s understanding of journalism and consequently their habitus. This process can lead individuals to believe that they do not possess the portrayed journalistic ideals or interpersonal power and that journalism is not a profession for them (Bourdieu, 1990). Thus, they might not even aspire to become journalists or might give up on their desired careers before attempting to pursue them (Threadgold, 2018). This dynamic is reinforced by a lack of role models and minimal contact with journalism. A 2019 Reuters report shows that many marginalized actors do not even consider journalism as a career, as it does not “run in the family,” nor have they ever met a journalist offline (Borchardt et al., 2019: 30). The same study finds that journalism is often viewed, especially within migrant communities, as neither honorable or financially viable, echoing experiences reported by journalists from working-class backgrounds (Vera-Zambrano and Powers, 2019).

Economic barriers further complicate the process for many marginalized individuals. Although entering the journalistic field does not technically require vast economic resources, access to legacy newsrooms increasingly depends on costly university degrees, relocating to metropolitan areas, which can come with increases in rent and living costs, and lengthy periods of underpaid or unpaid training (Borchardt et al., 2019; Nölleke et al., 2022). This shift privileges aspiring journalists with sufficient economic capital or familial support to endure years of precarious employment (Deuze and Witschge, 2018; Vera-Zambrano and Powers, 2019) and thus reproduces intersectional social inequalities (Collins and Bilge, 2020).

Social and cultural capital further play a crucial role in securing employment. Knowing the right people (Hummel et al., 2012) or possessing a (prestigious) degree, journalistic training or beat-specific knowledge increases one’s chances of entering the field successfully (Nölleke et al., 2022). This disadvantages others who might not be able to study, as they lack the support to navigate university, do not fulfill entry requirements or have care responsibilities.

The journalistic habitus and capital required to enter the profession are intertwined with the broader, overarching doxic belief system, or cultural domain of power, shaping the field. This system delineates what constitutes journalism, linking an actor’s “worthiness” to their background. For instance, whether an individual comes from an academic or a working-class family can shape their prospects, equipping them with more or less power and agency (Threadgold, 2018). Similarly, while a marginalized background—such as being Indigenous—may be valued during recruitment, research indicates that once hired, these journalists are often marginalized, exploited or rendered invisible due to their minority status and perceived lack of worth (Thomas and Nolan, 2024). Here, doxa determines whether an individual possesses the “right” habitus and resources to become a journalist, thereby shaping their ability to enter the profession. While some organizations might not be aware of potential hiring biases, unconsciously excluding disempowered actors from the field, others might use their disciplinary power to explicitly hire marginalized journalists with malintent. Research from Australia and Ethiopia indicates that employers might seek to benefit from Indigenous applicants’ or BIPoC’s “racial capital” (Leong, 2013: 2135; see also Lachover, 2022; Thomas and Nolan, 2024). At the same time, studies show that these marginalized applicants are expected to seamlessly adapt to the organization’s newsroom culture, thereby stipulating homogenization as a hiring criterion. Homogenization occurs when dominant journalists naturalize a certain doxic understanding of the journalistic profession and (unconsciously) project it onto more dominated actors as prerequisites for acceptance into the organization and the journalistic field (Bourdieu, 1990). For instance, an aspiring indigenous woman journalist in Australia was required to undergo voice training to modify her higher pitch and accent (Thomas and Nolan, 2024).

Actors with less symbolic power are also confronted with the question of whether they are willing to invest their time, efforts and physical and mental well-being into a profession in which they are structurally disadvantaged (Threadgold, 2018). While it is not necessarily made consciously, the resulting decision showcases the commitment of marginalized actors to be “taken in and by the game” (Bourdieu and Wacquant, 1992: 116). Despite a potential mismatch in habitus, doxa and the required capital (thus constituting a lack of interpersonal power), an actor’s illusio and aspiration to participate in the journalistic field can serve as drivers for change. For example, journalists of color in the UK who face racial discrimination critically engage with diversity policies—often leveraging alternative forms of capital such as gender or seniority—in an attempt to instigate change in the field (Douglas, 2022).

### Being a journalist

Once an actor, marginalized or not, enters the journalistic field, their career trajectories can vary. Whereas actors who match what is deemed valuable and worthy seem to move more effortlessly through their journalistic careers, actors experiencing a mismatch between individual predispositions and the demanded journalistic habitus or capital continue to struggle after entering the profession. Challenges emerge when journalists experience a clash between what they imagined the profession would be like (illusio) and what it is actually like (disillusion) (Nölleke et al., 2022) due to, for example, missing symbolic power.

Symbolic power in newsrooms can be exerted through defining the “rules of the game.” A prominent example is the normative idea of journalistic objectivity, which continues to permeate what journalistic work is deemed valuable. Journalistic objectivity assumes that a detached approach to observing and reporting on public life can compensate for an individual journalist’s influence on their news product (Tuchman, 1978). However, scholars have repeatedly pointed toward the “systematic blind-spots” (Schmidt, 2024: 548) of objectivity, as it tends to negate the lived realities and embodied knowledge that depart from the perception of an objective outlook. Moreover, studies show that objectivity is employed to justify the marginalization of certain perspectives and to exert symbolic violence under the cover of journalistic righteousness. For instance, research indicates that some editor-in-chiefs view women, disabled and/or (ethnic) minority journalists as too (emotionally) invested (Jones et al., 2021; Schmidt, 2024; Steiner, 2012), or their perspectives as too exotic or primitive (Thomas and Nolan, 2024), minimizing these journalists’ editorial freedom. Jones and colleagues (2021) argue that social justice for disabled people can only occur if they receive appropriate representation in the news, ultimately questioning the objectivity paradigm’s necessity to conceal the perspective from which journalists choose, produce and distribute an issue.

Power struggles and disillusionment appear not only in journalistic norms such as objectivity but also in editorial decision-making processes, such as news selection and news values. Here, research points to journalists’ shared gut feeling: how some news values are manifested as common sense and others are either orthodox and agreed upon, or heterodox and up for debate. The question of what is deemed orthodox or heterodox is the result of discursive negotiations in the field (Schultz, 2007). Disempowered journalists are once more at a disadvantage when they apply more heterodox or deviant news values, such as focusing on marginalized communities (Varma, 2023).

Disempowering practices also occur through assignment allocations within newsrooms. For instance, female journalists across continents are more often assigned to report on less prestigious soft news issues (North, 2016; Rao and Rodny-Gumede, 2020; Schultz, 2007), African American journalists describe having to report more often on issues of race (Shafer, 1993), and Australian Indigenous journalists discuss having to cover the “continued impacts of settler colonial violence” (Thomas and Nolan, 2024: 12). In contrast, the dominant actors in the newsroom experience more freedom in their topic selection and are not required to act as a proxy for the presumed communities they belong to (Douglas, 2022; North, 2016).

Hierarchical structures and promotion patterns in newsrooms have also been described as instances where symbolic violence can be exerted upon marginalized actors. In many regions, men traditionally hold powerful positions in newsrooms (e.g., editor-in-chief), while women and/or journalists of color are regularly excluded from authoritative positions, limiting their prospects to challenge unequal structures and dominant doxa (Govender and Muringa, 2025; Guo and Fang, 2023; Macupe, 2025). Newsrooms have been described as male-dominated “warzones” that reproduce sexism and racism, creating a strenuous environment for marginalized actors to navigate (Guo and Fang, 2023; Lucht and Batschelet, 2019). Overly masculine and/or ethnicity-specific approaches can reproduce sexist or racist ideologies within newsrooms, creating a “double burden” (Banjac, 2024b: 364) for marginalized journalists. This means that their safety and well-being is threatened both in newsrooms—through discriminating norms, values and practices (Banjac, 2024b; Govender and Muringa, 2025; Guo and Fang, 2023)—and society at large, as they are highly visible targets for censorship, (sexual) harassment and attacks by sources and audience members, as indicated by studies on female journalists in Pakistan, Mexico and Mozambique, among others (Jamil, 2020; Tsandzana, 2024; Valderrama, 2023).

Such tensions can lead marginalized journalists to engage in processes of conformity, assimilation, homogenization or code-switching to fit the dominant newsroom culture, thus consciously giving up some of their individual agency in exchange for more epistemic authority over their work (Bratcher and Cabosky, 2024). The pressure to adapt stems from the desire for recognition by journalistic peers (symbolic capital) but also from audience expectations to portray a certain journalistic image. This leaves little to no room for marginalized journalists to incorporate their diverging perspectives into reporting practices or editorial processes (Konieczna and Santa Maria, 2023).

Not all marginalized actors experience this struggle to belong equally. While research documents the mismatch between individuals’ habitus and the field’s structure (Beazer et al., 2025b; Douglas, 2022) , some actors actively capitalize on their marginalization, turning it into a strategic asset. Yet, intersectional power dynamics shape who can do this. For instance, white women may leverage gender to gain sympathy or privileges in newsrooms, whereas Black female journalists are often denied the same treatment (Macupe, 2025; see also Collins and Bilge, 2020). In such cases, actors such as white women may shift from being symbolically dominated (based on their gender) to exerting symbolic violence themselves (based on their race).

### Breaking away from journalism

While some marginalized journalists actively employ structural inequalities to occupy dominant positions in the field, others can feel seriously frustrated by them and might question their agency, losing their illusio to participate in journalism (Bourdieu, 1998). Consequently, some journalists may choose to leave the field, feeling disconnected from their original motivations and aspirations (Örnebring and Möller, 2018). Research across the globe indicates that women journalists leave the profession more frequently than men, often citing the prevalent “‘bloke’s club’ mentality” in newsrooms (Melki and Mallat, 2019: 33; see also North, 2012), which perpetuates gendered stereotypes (Guo and Fang, 2023; Rao and Rodny-Gumede, 2020). Furthermore, factors such as pay disparity, lack of promotions, short-term contracts, inadequate maternity leave or the “motherhood penalty,” overly long working hours and being confined to certain beats adversely shape women’s experiences in journalism (Melki and Mallat, 2019; North, 2016; Örnebring and Möller, 2018). Similarly, research suggests that marginalized journalists, such as female or Black journalists, report a lack of support, a sense of powerlessness and experiences of tokenism, contributing to their departure from newsrooms and the industry at large (Lachover, 2022; Mathews et al., 2023). This becomes particularly visible when examining the positions these marginalized journalists occupy in the newsroom hierarchy, with a majority holding low-level positions where they are unable to utilize their intersectional capital to effect change (Konieczna and Santa Maria, 2023). Thus, marginalized journalists’ habitus and accumulated capital oftentimes clash with the field’s structure, resulting in their leaving of journalism.

Such a clash between the personal and professional habitus can evoke emancipation through the hysteresis effect (Bourdieu, 1990), which could spark diversity initiatives and contribute to social change. However, research shows journalists lamenting a conscious erasure or exploitation of their lived experiences by those in power (Douglas, 2022; Melki and Mallat, 2019; Thomas and Nolan, 2024). Thus, there is a disconnect between news organizations’ commitments to diversity and the current realities that marginalized journalists face in newsrooms, ultimately resulting in journalists either adapting or leaving the field (Douglas, 2022; Jenkins and Finneman, 2018).

Of those that leave, some abandon journalism altogether, while others remain in the broader field of cultural production, creating and sharing content outside the constraints of traditional media. From the periphery, they challenge the journalistic doxa with heterodox beliefs and may establish alternative media outlets, such as feminist, LGBTQIA+, PoC and/or disability media, providing new perspectives and serving as alternatives or counterpublics to mainstream media (Beazer et al., 2025a; Maares and Hanusch, 2023).

## Where do we go from here?

The aim of this conceptual framework is to highlight the ways in which unequal distribution of symbolic power can shape the experiences of marginalized actors in the journalistic field within the context of their career trajectories. By tracing the potential development of journalists’ careers, we call for a reconsideration of current trends in diversity research, shifting the focus away from measuring how much “diversity” exists to instead exploring the conditions under which diversity is intersectionally constructed, contested and (un)consciously confined in journalism.

This framework is not case- or social context-dependent, but applicable across different journalistic fields globally, as it is grounded in the intersectional distribution and exertion of power in society. Thus, it serves as a map for future research to better grasp the intricate temporal (stages of the career) and spatial (field positions) intersectional power relations among diverse actors and their immediate surroundings, such as the newsroom, while recognizing the relational possibilities of interpersonal power within a given field (Collins and Bilge, 2020). Furthermore, this conceptual framework highlights the emancipatory potential for future media policy-making and greater newsroom diversity by pinpointing possible areas of contestation and oppression. It helps us to understand how diversity guidelines alone address persisting inequalities on a surface level, ignoring more subtle exertions of symbolic violence characterizing global journalistic and newsroom cultures that limit individual agency and collective action.

This is not to say that explicit diversity guidelines in hiring procedures cannot contribute to reaching a critical mass to instigate change (Assmann and Eckert, 2024). Nevertheless, simply increasing the presence of marginalized journalists within newsrooms will not automatically alleviate unequal power hierarchies (Lünenborg and Weiß, 2025), as seen in regards to women and Black people in South African newsrooms (Rao and Rodny-Gumede, 2020). Rather, current norms, such as the objectivity paradigm and working practices shaping story selection, assignment allocations and overall working conditions, need to be reevaluated to manifest long-lasting diversity within the journalistic field. Hence, disempowered journalists already present in newsrooms could take on leading roles. As Bourdieu (2000: 234) argued, a mismatch between individual and field habitus and its resulting tensions (hysteresis effect) can contribute to social change and carry emancipatory potential, where individuals collectively contest power structures and taken-for-granted beliefs in a field (Masquelier, 2019). This contestation of unequal power stratifications is not limited to actors within the journalistic field but can also occur from its periphery. Peripheral actors, individuals and collectives alike can challenge and potentially improve (in an agonistic approach) or attempt to deconstruct and destroy journalism’s paradigmatic position in society (in an antagonistic approach) (Eldridge, 2019).

While we aimed to include a wide variety of identity markers in our examples for this conceptual framework, there are many more intersectional characteristics deserving equal attention in research. Furthermore, as two European, able-bodied, well-educated and white women from a relatively well-acknowledged university, we are aware of our systematic blind-spots in researching the topic of diversity. However, we believe that combining field theory and its focus on power struggles with intersectionality provides a fruitful framework to study these further.

Going forward, empirical research should address the underlying and intersectional mechanisms of symbolic violence more closely to envision solutions and innovative practices that reshape the journalistic field into a more inclusive and socially just space, a key theme within intersectionality (Collins and Bilge, 2020). While a rich body of research exists on how symbolic violence is exerted upon individuals with specific identity markers (e.g., race or gender), research on more intersectional approaches to inequalities in journalism is lacking, except for a few notable exemptions (Banjac, 2024a; Lünenborg and Weiß, 2025). Furthermore, scholarship should focus on those situated within the periphery of the field, evaluating their effects on journalism and their definition of journalistic symbolic capital. Lastly, it is also important to consider that journalism does not happen in a vacuum but is relationally constituted in correspondence with other fields. Thus, Journalism Studies as an academic field must reflect more consciously on the ways it contributes to the reproduction of symbolic power and violence, be it through who we study, what forms of journalism we consider legitimate or our curricula in journalism education (Mohammed, 2022).

## Data Availability

There is not dataset attached to this research.*

## Note

1. We intentionally chose the term “marginalized” instead of “minority,” as the marginalized are not necessarily minorities (e.g., women are not a minority but tend to be marginalized in many societies).

## References

[bibr1-14648849261416555] Ahmed S (2007) “You end up doing the document rather than doing the doing”: diversity, race equality and the politics of documentation. Ethnic and Racial Studies 30(4): 590–609.

[bibr2-14648849261416555] Assmann K Eckert S (2024) Are women journalists in leadership changing work conditions and newsroom culture? Journalism 25(3): 565–584.

[bibr3-14648849261416555] Awad-Cherit I (2008) Cultural diversity in the news media: a democratic or a commercial need? Javnost - The Public 15(4): 55–72.

[bibr4-14648849261416555] Banjac S (2024a) Intersectionality and standpoint epistemology in journalism studies. In: Badr H Wilkins K (eds) Critical Communication Research with Global Inclusivity. New York, NY: Routledge.

[bibr5-14648849261416555] Banjac S (2024b) Safety of journalists. In: Eldridge SA Cheruiyot D Banjac S , et al. (eds) The Routledge Companion to Digital Journalism Studies. 2nd edition. London: Routledge, 358–367.

[bibr6-14648849261416555] Beazer A Walter S Eldridge SA , et al. (2025a) On the margins: exploring minority news media representations of women during the COVID-19 pandemic. Digital Journalism 13(6): 1–20.

[bibr7-14648849261416555] Beazer A Walter S Eldridge Ii SA (2025b) Space for solidarity? Studying women journalists’ experiences of covering minoritized groups. Journalism Practice 1–21.

[bibr8-14648849261416555] Bodinger-de Uriarte C Valgeirsson G (2015) Institutional disconnects as obstacles to diversity in journalism in the United States. Journalism Practice 9(3): 399–417.

[bibr9-14648849261416555] Borchardt A Lück J Kieslich S , et al. (2019) Are journalists today’s coal miners? The struggle for talent and diversity in modern newsrooms: a study on journalists in Germany, Sweden, and the United Kingdom. UK: Report, Reuters Institute for the Study of Journalism.

[bibr10-14648849261416555] Botma GJ (2012) The language(s) of failure? Unequal access to journalism education and training at a former whites-only Afrikaans university in South Africa. Journalism & Mass Communication Educator 67(1): 26–44.

[bibr11-14648849261416555] Bourdieu P (1977) Outline of a Theory of Practice (Trans. Nice R). 1st edition. Cambridge, London, New York, Melbourne: Cambridge University Press.

[bibr12-14648849261416555] Bourdieu P (1984) Distinction: A Social Critique of the Judgement of Taste. Cambridge, MA: Harvard University Press.

[bibr81-14648849261416555] Bourdieu P (1986) The Forms of Capital. In: Richardson J. (ed). *Handbook of Theory and Research for the Sociology of Education* . Greenwood, 241–258.

[bibr13-14648849261416555] Bourdieu P (1989) Social space and symbolic power. Sociological Theory 7(1): 14–25.

[bibr14-14648849261416555] Bourdieu P (1990) The Logic of Practice. Stanford University Press.

[bibr15-14648849261416555] Bourdieu P (1993) The Field of Cultural Production: Essays on Art and Literature. Cambridge: Polity Press.

[bibr16-14648849261416555] Bourdieu P (1998) Practical Reason: On the Theory. Stanford, CA: Stanford Univ. Press.

[bibr17-14648849261416555] Bourdieu P (2000) Pascalian Meditations. Standford. Stanford Univ. Press.

[bibr18-14648849261416555] Bourdieu P Wacquant L (1992) An Invitation to Reflexive Sociology. Chicago: University of Chicago Press.

[bibr19-14648849261416555] Bratcher TR Cabosky J (2024) Code-switching candidates: strategic communication, podcasts, and cultural and racial politics. Atlantic Journal of Communication 32(1): 1–15.

[bibr20-14648849261416555] Byerly CM Sha B-L Grant RL , et al. (2023) The versatility of intersectionality in journalism and mass communication research. Journalism & Mass Communication Quarterly 100(2): 249–263.

[bibr21-14648849261416555] Carter AW Ferrucci P (2024) The professional backstaging of diversity in journalism. Journal of Communication 74(3): 212–224.

[bibr22-14648849261416555] Carter C Steiner L Allan S (eds) (2019) Journalism, Gender and Power. London; New York: Routledge, Taylor & Francis Group.

[bibr23-14648849261416555] Collins PH Bilge S (2020) Intersectionality. 2nd edition. Cambridge: Polity Press.

[bibr25-14648849261416555] Crenshaw K (1989) Demarginalizing the intersection of race and sex: a black feminist critique of antidiscrimination doctrine, feminist theory and antiracist politics. University of Chicago Legal Forum 1989(1): 8.

[bibr26-14648849261416555] Crenshaw K (1991) Mapping the margins: intersectionality, identity politics, and violence against women of color. Stanford Law Review 43(6): 1241–1299.

[bibr27-14648849261416555] Deuze M Witschge T (2018) Beyond journalism: theorizing the transformation of journalism. Journalism 19(2): 165–181. 10.1177/1464884916688550 PMC5777551 29417952

[bibr28-14648849261416555] Djerf-Pierre M (2007) The structure and logic of the field in the twentieth century. Nordicom Review 28: 81–104.

[bibr29-14648849261416555] Douglas O (2022) The media diversity and inclusion paradox: experiences of black and brown journalists in mainstream British news institutions. Journalism 23(10): 2096–2113.

[bibr30-14648849261416555] Eldridge SA (2019) Where do we draw the line? Interlopers, (ant)agonists, and an unbounded journalistic field. Media and Communication 7(4): 8–18.

[bibr31-14648849261416555] Govender G Muringa TP (2025) “Women will never be equal to men”: examining women journalists’ experiences of patriarchy and sexism in South Africa. Journalism and Media 6(1): 27.

[bibr32-14648849261416555] Guo J Fang K (2023) Where are the missing girls? Gender inequality, job precarity, and journalism students’ career choices in China. Journalism 24(10): 2099–2117.

[bibr33-14648849261416555] Hanitzsch T Vos T (2017) Journalistic roles and the struggle over institutional identity: the discursive constitution of journalism: journalistic roles and institutional identity. Communication Theory 27(2): 115–135.

[bibr35-14648849261416555] Hovden JF (2008) Profane and Sacred: A Study of the Norwegian Journalistic Field. Bergen: University of Bergen.

[bibr36-14648849261416555] Hummel R Kirchhoff S Prandner D (2012) “We used to be queens and now we are slaves”: working conditions and career strategies in the journalistic field. Journalism Practice 6(5–6): 722–731.

[bibr37-14648849261416555] Jamil S (2020) Suffering in silence: the resilience of Pakistan’s female journalists to combat sexual harassment, threats and discrimination. Journalism Practice 14(2): 150–170.

[bibr38-14648849261416555] Jenkins J Finneman T (2018) Gender trouble in the workplace: applying Judith Butler’s theory of performativity to news organizations. Feminist Media Studies 18(2): 157–172.

[bibr39-14648849261416555] Jones C Changfoot N Johnston K (2021) Representing disability, d/deaf, and mad artists and art in journalism: identifying ableist fault lines and promising crip practices of representation. Studies in Social Justice 15(2): 307–333.

[bibr40-14648849261416555] Kanter RM (1997) Men and Women of the Corporation. New York, NY: Basic Books.

[bibr41-14648849261416555] Konieczna M Santa Maria E (2023) “I can’t be neutral or centrist in a debate over my own humanity”: a study of disagreements between journalists and editors, and what they tell us about objectivity. Journalism Studies 24(15): 1–18.

[bibr42-14648849261416555] Lachover E (2022) Ethiopian-Israeli women working in mainstream journalism: linking Ethiopian, black and Israeli identities through news-making. Journalism 23(10): 2041–2059.

[bibr43-14648849261416555] Leong N (2013) Racial capitalism. Harvard Law Review 126(8): 2151–2226.

[bibr44-14648849261416555] Lindell J Kas AD (2024) Media practice and class-making: the anticipation of stigma and the cultural middle-class habitus. Media, Culture & Society 46(1): 21–37.

[bibr45-14648849261416555] Lucht T Batschelet K (2019) “That was what I had to use”: social and cultural capital in the careers of women broadcasters. Journalism & Mass Communication Quarterly 96(1): 194–214.

[bibr46-14648849261416555] Lünenborg M Weiß A-N (2025) XXVII. Diversität im Journalismus. In: Hanitzsch T Loosen W Sehl A (eds) Journalismusforschung. Baden-Baden. Nomos Verlagsgesellschaft mbH & Co. KG, 547–564.

[bibr47-14648849261416555] Maares P Hanusch F (2022) Interpretations of the journalistic field: a systematic analysis of how journalism scholarship appropriates Bourdieusian thought. Journalism 23: 736–754.

[bibr48-14648849261416555] Maares P Hanusch F (2023) Understanding peripheral journalism from the boundary: a conceptual framework. Digital Journalism 11: 1–22.

[bibr49-14648849261416555] Macupe B (2025) What It Means to Be a Black African Woman Navigating South African Newsrooms. UK: Journalist Fellowship Paper, Reuters Institute for the Study of Journalism.

[bibr50-14648849261416555] Malik S (2014) Diversity, broadcasting and the politics of representation. In: Horsti K Hultén G Titley G (eds) National Conversations: Public Service Media and Cultural Diversity in Europe. Bristol: Intellect Books, 40–41.

[bibr51-14648849261416555] Masquelier C (2019) Bourdieu, Foucault and the politics of precarity. Distinktion: Journal of Social Theory 20(2): 135–155.

[bibr52-14648849261416555] Mathews N Bélair-Gagnon V Carlson M (2023) “Why I quit journalism”: former journalists’ advice giving as a way to regain control. Journalism 24(1): 62–77.

[bibr53-14648849261416555] McQuail D (1992) Media Performance: Mass Communication and the Public Interest. London; Newbury Park, CA: Sage Publications.

[bibr54-14648849261416555] Melki J Mallat S (2019) When Arab women (and men) speak: struggles of women journalists in a gendered news industry. In: Carter C Steiner L Allan S (eds) Journalism, Gender and Power. London: Routledge, 33–48.

[bibr55-14648849261416555] Mirza HS (2006) Transcendence over diversity: black women in the academy. Policy Futures in Education 4(2): 101–113.

[bibr56-14648849261416555] Mohammed WF (2022) Dismantling the Western canon in media studies. Communication Theory 32(2): 273–280.

[bibr57-14648849261416555] Moore R (2014) Capital. In: Grenfell MJ (ed) Pierre Bourdieu: Key Concepts. 2nd edition. New York: Routledge, 98–113.

[bibr58-14648849261416555] Nölleke D Maares P Hanusch F (2022) Illusio and disillusionment: expectations met or disappointed among young journalists. Journalism 23(2): 320–336.

[bibr59-14648849261416555] Nölleke D Hanusch F Maares P (2023) The ambivalence of recognition: how awarded journalists assess the value of journalism prizes. Journalism 24(7): 1406–1423.

[bibr60-14648849261416555] North L (2012) “Blokey” newsrooms still a battleground for female journalists. Australian Journalism Review 34(2): 57–70.

[bibr61-14648849261416555] North L (2016) The gender of “soft” and “hard” news: female journalists’ views on gendered story allocations. Journalism Studies 17(3): 356–373.

[bibr62-14648849261416555] Örnebring H Möller C (2018) In the margins of journalism: gender and livelihood among local (ex-)journalists in Sweden. Journalism Practice 12(8): 1051–1060.

[bibr63-14648849261416555] Rao S Rodny-Gumede Y (2020) Gazing past the glass ceiling: indian and South African female journalists’ perceptions of their role and power in the newsroom. Global Media and Communication 16(1): 57–74.

[bibr64-14648849261416555] Ross Arguedas A Mukherjee M Nielsen RK (2024) Women and leadership in the news media 2024: evidence from 12 markets. Reuters Institute for the Study of Journalism. Available at: https://reutersinstitute.politics.ox.ac.uk/women-and-leadership-news-media-2024-evidence-12-markets (accessed 12 July 2024).

[bibr65-14648849261416555] Schmidt TR (2024) Challenging journalistic objectivity: how journalists of color call for a reckoning. Journalism 25(3): 547–564.

[bibr66-14648849261416555] Schubert JD (2014) Suffering/symbolic violence. In: Grenfell MJ (ed) Pierre Bourdieu: Key Concepts. 2nd edition. New York: Routledge, 179–194.

[bibr67-14648849261416555] Schultz I (2007) The journalistic gut feeling: Journalistic doxa, news habitus and orthodox news values. Journalism Practice 1(2): 190–207.

[bibr68-14648849261416555] Shafer R (1993) What minority journalists identify as constraints to full newsroom equality. Howard Journal of Communications 4(3): 195–208.

[bibr69-14648849261416555] Steiner L (2012) Failed theories: explaining gender difference in journalism. Review of Communication 12(3): 201–223.

[bibr70-14648849261416555] Sterne J (2003) Bourdieu, technique and technology. Cultural Studies 17(3–4): 367–389.

[bibr71-14648849261416555] Thomas A Nolan D (2024) “It’s trauma on a deadline”: change, continuity and harm after the “racial reckoning.”. Digital Journalism: 1–19.

[bibr72-14648849261416555] Thomson P (2014) Field. In: Grenfell MJ (ed) Pierre Bourdieu: Key Concepts. 2nd edition. New York: Routledge, 65–82.

[bibr73-14648849261416555] Threadgold S (2018) Bourdieu is not a determinist: illusio, aspiration, reflexivity and affect. In: Stahl G Wallace D Burke C , et al. (eds) International Perspectives on Theorizing Aspirations: Applying Bourdieu’s Tools. London: Bloomsbury Academic, 36–50.

[bibr74-14648849261416555] Topić M Bruegmann C (2021) “The girls at the desk”: timeless blokishness in the newsroom culture in the British press? Journalism Studies 22(1): 77–95.

[bibr75-14648849261416555] Tsandzana D (2024) Being a woman-journalist in a polarized context in Mozambique. In: Tshuma LA Matsilele T Msimanga MJ , et al. (eds) Patterns of Harassment in African Journalism. 1st edition. London: Routledge, 28–48.

[bibr76-14648849261416555] Tuchman (1978) Making News A Study in the Construction of Reality. New York, NY: Free Press.

[bibr77-14648849261416555] Valderrama YZ (2023) Women journalists in Mexico. In: Mutsvairo B Bebawi S Borges-Rey E (eds) The Routledge Companion to Journalism in the Global South. 1st edition. London: Routledge, 378–387.

[bibr78-14648849261416555] Varma A (2023) Solidarity reporting on marginalization: a grounded alternative to monitorial reporting’s emphasis on officials. Journalism Practice 19(1): 1–17.

[bibr79-14648849261416555] Vera-Zambrano S Powers M (2019) The roots of journalistic perception. In: Polson E Clark LS Gajjala R (eds) The Routledge Companion to Media and Class. 1st edition. Routledge, 157–167.

[bibr80-14648849261416555] Webb S Burke PJ Nichols S , et al. (2017) Thinking with and beyond Bourdieu in widening higher education participation. Studies in Continuing Education 39(2): 138–160.

